# Changes in the gastric enteric nervous system and muscle: A case report on two patients with diabetic gastroparesis

**DOI:** 10.1186/1471-230X-8-21

**Published:** 2008-05-30

**Authors:** Pankaj J Pasricha, Nonko D Pehlivanov, Guillermo Gomez, Harsha Vittal, Matthew S Lurken, Gianrico Farrugia

**Affiliations:** 1 Division of Gastroenterology, Department of Medicine, Stanford University School of Medicine, Stanford, CA, USA; 2 Enteric Neuromuscular Disorders and Pain Laboratory, Division of Gastroenterology, Department of Medicine, University of Texas Medical Branch, Galveston, TX, USA; 3 Department of Surgery, University of Texas Medical Branch, Galveston, TX, USA; 4 Enteric NeuroScience Program, Mayo Clinic College of Medicine, Rochester, MN, USA

## Abstract

**Background:**

The pathophysiological basis of diabetic gastroparesis is poorly understood, in large part due to the almost complete lack of data on neuropathological and molecular changes in the stomachs of patients. Experimental models indicate various lesions affecting the vagus, muscle, enteric neurons, interstitial cells of Cajal (ICC) or other cellular components. The aim of this study was to use modern analytical methods to determine morphological and molecular changes in the gastric wall in patients with diabetic gastroparesis.

**Methods:**

Full thickness gastric biopsies were obtained laparoscopically from two gastroparetic patients undergoing surgical intervention and from disease-free areas of control subjects undergoing other forms of gastric surgery. Samples were processed for histological and immunohistochemical examination.

**Results:**

Although both patients had severe refractory symptoms with malnutrition, requiring the placement of a gastric stimulator, one of them had no significant abnormalities as compared with controls. This patient had an abrupt onset of symptoms with a relatively short duration of diabetes that was well controlled. By contrast, the other patient had long standing brittle and poorly controlled diabetes with numerous episodes of diabetic ketoacidosis and frequent hypoglycemic episodes. Histological examination in this patient revealed increased fibrosis in the muscle layers as well as significantly fewer nerve fibers and myenteric neurons as assessed by PGP9.5 staining. Further, significant reduction was seen in staining for neuronal nitric oxide synthase, heme oxygenase-2, tyrosine hydroxylase as well as for c-KIT.

**Conclusion:**

We conclude that poor metabolic control is associated with significant pathological changes in the gastric wall that affect all major components including muscle, neurons and ICC. Severe symptoms can occur in the absence of these changes, however and may reflect vagal, central or hormonal influences. Gastroparesis is therefore likely to be a heterogeneous disorder. Careful molecular and pathological analysis may allow more precise phenotypic differentiation and shed insight into the underlying mechanisms as well as identify novel therapeutic targets.

## Background

Long-standing and poorly-controlled diabetes may result in many disturbances in gastrointestinal function of which gastroparesis is one of the more common. In tertiary referral centers gastroparesis is seen in 20–55% of patients with Type I and up to 30% of patients with type II diabetes [1]. The pathogenesis of gastroparesis is poorly understood, in part because of a lack of prospective comprehensive studies of gastric pathology in these patients. Further, while animal models suggest that the pathogenesis of diabetic gastroparesis is likely to be multifactorial involving the vagus, intrinsic nerves, interstitial cells and smooth muscle of the stomach, the relative importance of these elements in explaining variations in the clinical presentation and natural history of patients remains unknown [2]. Here we report the first systematic analysis of gastric pathology in two humans with diabetic gastroparesis, using histological and immunohistochemical techniques. Our results emphasize the heterogeneity of this syndrome and provide several hypotheses for future studies.

## Methods

### Patients

Both patients reported here were referred to the University of Texas Medical Branch (UTMB) with a diagnosis of diabetic gastroparesis and refractory symptoms. The patients consented to participate in a prospective study of full thickness gastric biopsies obtained laparoscopically at the time of surgical placement of a jejunal tube or gastric electrical stimulator placement. All ages reported here are at the time of the gastric biopsy.

### Full thickness gastric biopsy

Full thickness pieces of tissue (1 × 2 cm in size) were taken by the surgeon (GG) above the level of the incisura in the anterior wall of the mid-body of the stomach. Control specimens (n = 2) were obtained from the corresponding area in non-diabetic obese patients undergoing gastric bypass surgery by the same surgeon.

### Pathological and immunohistochemical examination of tissue

For immunohistochemistry studies, biopsy specimens were fixed in 4% paraformaldehyde (Sigma-Aldrich, St. Louis, MO) in 1× phosphate buffer solution, rinsed in 1× phosphate buffered saline (PBS) and immersed overnight in 1× PBS solution containing 30% sucrose (Sigma-Aldrich, St. Louis, MO). All incubations were completed at 4°C. The biopsies were cut in cross section and frozen in Tissue-Tek OCT compound (Electron Microscopy Sciences, Hatfield, PA). 12 μm thick cryostat sections were cut. A standard H&E and Trichrome stain protocol was completed on the sections. The presence of fibrosis was assessed using Masson's trichrome stain. For immunohistochemistry six different antibodies were used following the same protocol outlined below. The antibodies used were a polyclonal rabbit anti-human protein gene product 9.5 (PGP 9.5) (1:2000, AbD Serotec, Oxford, UK), polyclonal rabbit anti-human neuronal nitric oxide synthase (nNOS) (1:4000, Chemicon, Temecula, CA), polyclonal sheep anti-rat tyrosine hydroxylase (TH) (1:200), polyclonal rabbit anti-substance P (1:1200, Chemicon, Temecula, CA), rabbit anti-human heme oxygenase II (HO-2) (1:10,000, kind gift from Dr. Snyder, Johns Hopkins University, Baltimore, MA), polyclonal rabbit anti-human c-Kit (2 ng/μl, MBL, Nagoya, Japan). The slides were first rinsed twice in 1× PBS followed by a blocking step for non-specific secondary antibody binding by incubating the tissue with a 1× PBS, 10% normal donkey serum (NDS; Jackson ImmunoResearch Lab, Inc., West Grove, PA) and 0.3% Triton ×-100 (Pierce, Rockford, IL) solution for one hour. The appropriate primary antibody was then diluted in 1× PBS, 5% NDS, 0.3% Triton ×-100 and incubated overnight at 4°C. A Cy3 conjugated donkey anti-rabbit secondary antibody or a Cy3 conjugated donkey anti-sheep secondary antibody (Jackson ImmunoResearch Lab, Inc., West Grove, PA) diluted in 1× PBS, 2.5% NDS, 0.3% Triton ×-100 solution was used to visualize the immuno-positive structures.

### Institutional approval

This study was approved by the Institutional Review Board of the University of Texas Medical Branch and the Mayo Clinic College of Medicine. Informed consent was obtained from all patients and controls.

## Results

### Clinical presentations

#### Case 1

This was a 37 year old white female who developed type 1 diabetes about 3 years prior to the biopsy. Gastroparetic symptoms of nausea and vomiting were present for approximately the same duration. Despite various therapies, she continued to have severe nausea and vomiting, along with weight loss. However, her diabetes remained well controlled throughout with a HbA1c of 6.2% at initial presentation.

#### Case 2

This was a 32 year old white female with a history of type I diabetes for about a decade. The diabetes was relatively difficult to control with numerous episodes of diabetic ketoacidosis and frequent hypoglycemic episodes. At the time of presentation her HbA1c ranged between 6.6 to 7%. For more than a year prior to placement of a gastric electrical stimulator, the patient had intractable symptoms of nausea, vomiting and pain with frequent hospitalizations.

Both patients had radiological and/or scintigraphic evidence of severely delayed gastric emptying. Although formal testing of the autonomic nervous system was not carried out, both patients also had resting tachycardia when they were relatively well, in the absence of overt dehydration. Finally, the long-term (> 1 year) response to gastric electrical stimulation was markedly different with patient 1 reporting a more than 50% improvement in symptoms as compared with baseline while patient 2 experienced no significant change in her clinical course.

### Pathological changes in the stomach

#### Assessment of fibrosis (Figure 1)

**Figure 1:**
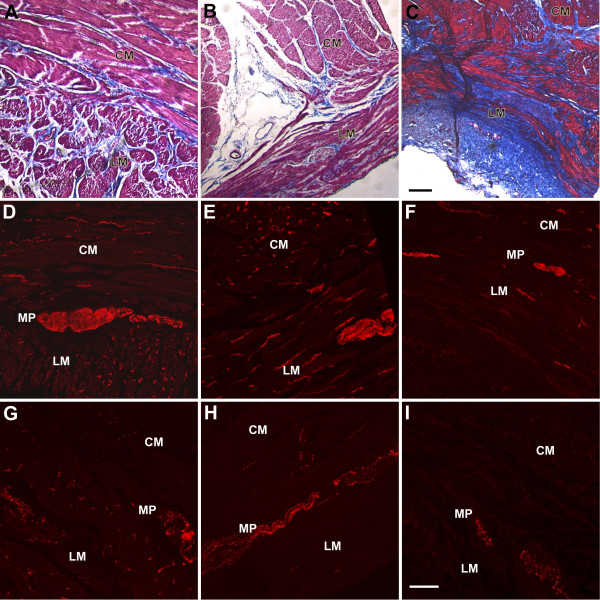
**Staining for fibrosis and for neuronal structures**. Panels A-C. Trichrome staining. The control (A) and sections from case 1 (B) showed no increase in fibrosis, whereas sections from case 2 (C) showed an increase in fibrosis in both muscle layers and along the myenteric plexus. Scale bar 200 μm. Panels D-F. PGP 9.5 immunoreactivity. PGP9.5 immunoreactivity as a marker for neuronal structures was normal in control (D) and in case 1 (E). There was a decrease in PGP 9.5 immunoreactivity in both the muscle layers and the myenteric plexus in sections from case 2 (F) suggesting a loss of neuronal structures. Panels G-I. Tyrosine hydroxylase immunoreactivity. Tyrosine hydroxylase immunoreactivity was normal in the control (G). Sections from case 1 (H) showing normal tyrosine hydroxylase immunoreactivity around the myenteric plexus with a lsight decrease in tyrosine hydroxylase immunoreactivity in the muscle layers. Sections from case 2 (I) showed a marked loss of tyrosine hydroxylase immunoreactivity in all regions suggesting a loss of extrinsic nerve fibers. Scale bar 100 μm. Circular muscle (CM), myenteric plexus (MP), longitudinal muscle (LM).

##### Case 1

Sections from the patient with well controlled diabetes showed a normal staining pattern with no evidence of increased fibrosis in the muscle layers.

##### Case 2

In contrast, sections from the tissue obtained from the patient with poorly controlled diabetes showed a marked increase in fibrosis in both muscle layers and around the myenteric plexus.

#### Enteric nerves (Figure 1)

The number of nerve cell bodies and fibers was assessed using antibodies against the pan-neuronal maker PGP 9.5.

##### Case 1

The number of nerve fibers in the circular and longitudinal muscle layers was normal in sections from the patient with well controlled diabetes when compared to the controls.

##### Case 2

There was a 40–50% decrease in the number of nerve fibers in the patient with poorly controlled diabetes as compared with controls.

#### Changes in the expression of neurotransmitters and related proteins (Figure 2)

**Figure 2:**
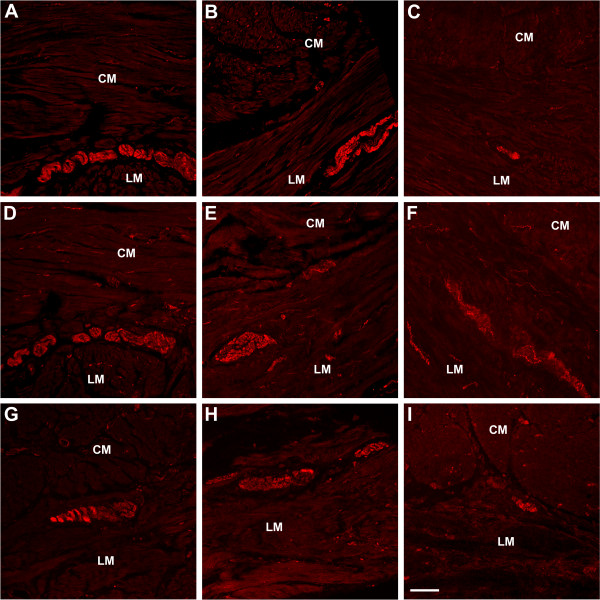
**Immunoreactiivty for nNOS, SP and HO2 as markers for inhibitory and excitatory nerve cell bodies and fibers**. Panels A-C. Normal immunoreactivity for nNOS in control (A) and case 1 (B). nNOS immunoreactivity was markedly decreased in case 2 (C). Panels D-F. Substance P expression was normal in control (D), case 1 (E), and case 2 (F). Panels G-I. Heme Oxygenase II (HO-2) immunoreactivity was normal in control (G) and case 1 (H). Sections from Case 2 (I) showed a loss of HO-2 immunoreactivity which together with the decrease in nNOS immunoreactivty suggests a loss of inhibitory input ot smooth muscle. Scale bar 100 μm. Circular muscle (CM), longitudinal muscle (LM).

Experiments were subsequently directed to determine if the loss of nerve fibers seen was specific to a particular subset of nerve fibers. Antibodies against tyrosine hydroxylase (TH) were used to immunolabel extrinsic nerve fibers. Antibodies against substance P (SP) and neuronal nitric oxide synthase (nNOS) were used to immunolabel excitatory nerves and inhibitory nerves respectively. Antibodies to heme oxygenase-2 (HO2) were used to label nerves and ICC that contain heme oxygenase, the enzyme that gives rise to carbon monoxide, known to regulate neurotransmission and smooth muscle membrane potential [3,4].

##### Case 1

No changes in expression of SP, nNOS, H02 (Figure 2) were noted in sections from the patient with well controlled diabetes when compared to controls. There was a slight decrease in the number of TH positive fibers in the muscle layers in this patient; however, TH immunoreactivity around ganglia was normal, suggesting an intact extrinsic innervation to the intrinsic nervous system (Figure 1).

##### Case 2

No significant changes were seen with the SP antibody but immunolabeling for nNOS, H02 (Figure 2) and TH (Figure 1) was decreased (by 40–50%) in the patient with poorly controlled diabetes.

### Interstitial cells of Cajal

Human gastric ICC are of two major types- those that are scattered throughout the stomach in both layers of muscle (ICC-IM) and those that are associated with the myenteric plexus (ICC-MY) in the body and antrum, but not in the more proximal stomach. In this study, ICC were immunolabeled using an antibody to c-Kit. ICC-MY were not visualized in either patient nor in control tissue in this region of the stomach.

#### Case 1

**Figure 3:**
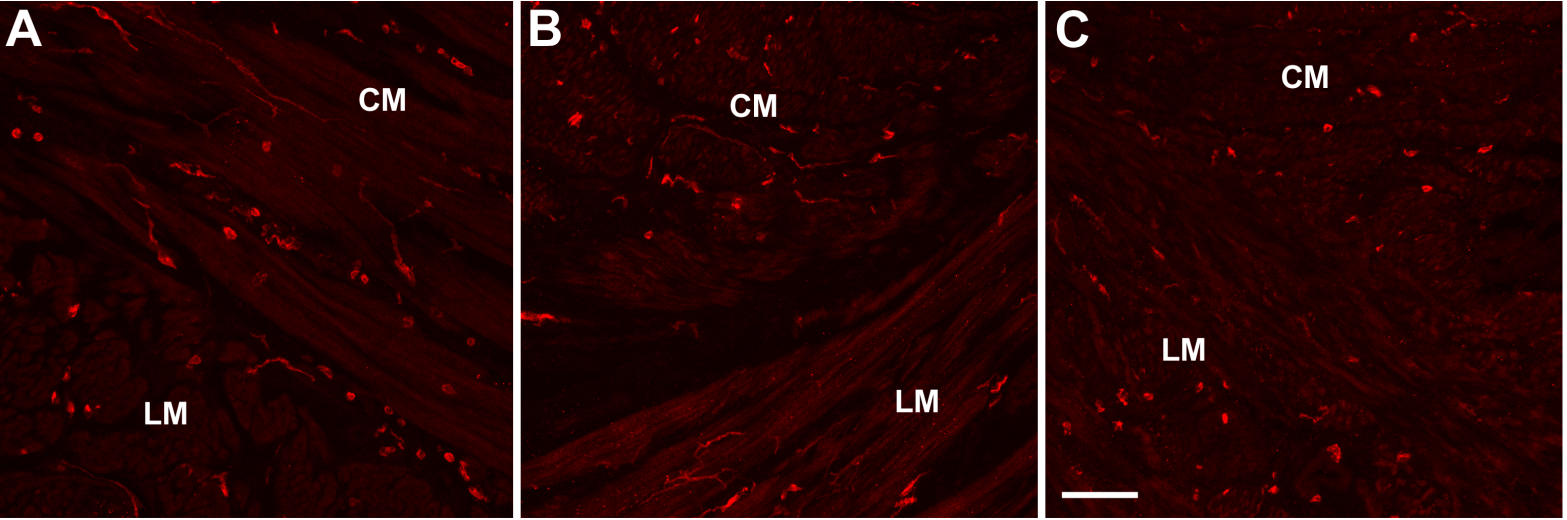
**c-Kit expression as a marker for interstitial cells of Cajal**. Control (A) and case 1 (B) showed normal c-Kit immunoreactivity while in case 2 (C) there was a loss of c-Kit immunoreactivity suggesting a decreased number of ICC. Scale bar 100 μm. Circular muscle (CM), longitudinal muscle (LM).

Distribution of ICC-IM was normal in the patient with well controlled diabetes with normal numbers of ICC (Figure 3).

#### Case 2

In the patient with poorly controlled diabetes the number of c-Kit positive ICC-IM was reduced in both muscle layers (by 30–40%, Figure 3).

## Discussion

Despite its existence as a clinical syndrome for more than half-a-century [5], the pathogenesis of diabetic gastroparesis remains unknown. Studies on experimental models of diabetes have been relatively narrowly focused on the interests of individual laboratories and indicate the involvement of several cell types (neurons, ICC, muscle), as reviewed recently [2]. The two most consistent findings appear to involve changes in neuronal nNOS expression and ICC. However, a unifying theme or hypothesis for these findings has still not emerged in part because of the inability to validate them in humans, in whom access to the deeper intramural structures generally requires surgery. The few studies that have obtained gastric tissue in this manner were generally done in an era when sophisticated imaging and molecular technologies did not exist and have produced results that are inconsistent at best. We report a systematic analysis of the gastric enteric nervous system and muscle of two patients with diabetic gastroparesis as compared with corresponding tissues obtained from obese controls. Striking changes were seen in one patient; these included a significant degree of fibrosis involving the muscle layer, which probably reflects the consequences of severe and perhaps repetitive tissue injury from metabolic, vascular or other complications of diabetes.

These changes are in keeping with a previous report showing severe collagen deposition and fibrosis in 4 patients with refractory diabetic gastroparesis [6,7]. On the other hand, another study of 16 patients with long-standing diabetes (five of whom had gastroparesis) showed no abnormalities in the smooth muscle (or other structures) [8]. Further, neither of these studies found any significant changes in the myenteric plexus, which is in contrast to our findings in Case 2, where a major reduction in myenteric neuronal staining was seen along with a reduction in markers for inhibitory neurotransmitters. Although we did not study markers of apoptosis or cell death in this study, the finding of decreased PGP9.5 labeling suggests that these changes reflect a degree of neuronal loss and not simply merely loss of expression of the synthetic enzyme or neurotransmitter. However, evidence for actual gastric neuronal loss in diabetes has not been reported in either animals or patients [2], although a single case study of a patient with idiopathic gastroparesis revealed markedly reduced numbers of PGP 9.5-positive myenteric neurons [9].

Nitric oxide is one of the most important neurotransmitters for the maintenance of normal gastric motility and there is considerable experimental literature implicating a role for its loss in the pathogenesis of delayed gastric emptying [10-19]. Most recently, a study of 36 male diabetic patients undergoing gastric resection showed significant loss of nNOS as well as SP expression; however, there was no information on whether these patients were symptomatic or had impaired gastric motility [20]. In our patient with poorly controlled diabetes, the reduction in overall neuronal staining was accompanied by a significant loss of nNOS, but not SP expression, suggesting relatively specific injury to inhibitory neurons. In this context, our study is also the first to report changes in HO-2, the constitutive form of heme oxygenase that is largely responsible for production of carbon monoxide, another endogenous gaseous neuromodulator of considerable importance in the inhibitory control of gastrointestinal motility [3,21-25]. The combined loss of both inhibitory molecules thus is expected to cause a profound disturbance of gastric physiology as was seen in our patient (Case 2). This case also illustrates the differences and similarities between changes in diabetic gastropathy as compared with diabetic enteropathy. The latter is also associated with loss of nNOS and TH expression but by contrast, presents with increased expression of SP [26], suggesting regional variations in the effects of diabetes on the enteric nervous system.

Finally, we also looked at changes in ICC using c-Kit as a marker. ICC play several critical roles in gastrointestinal physiology including setting the pacemaker rhythm, and participation in neurotransmission and mechanosensitivity [21,27-29]. Both in vivo and in vitro studies of experimental diabetes report ICC depletion, perhaps secondary to loss of insulin/IGF and/or nitric oxide or other, as yet undetermined, survival factors [7,30-32]. Our findings of significant loss of c-Kit expression in Case 2 are consistent with other changes noted in this patient as well as with emerging reports in humans. Thus, four out of nine patients with refractory diabetic gastroparesis were found to have significant loss of ICC [33]. A reduction in c-Kit staining was also noted in the study on diabetic patients undergoing gastric resection for cancer [20].

Our study is important as it provides a rare insight into the pathological as well as molecular changes of potential importance in the pathogenesis of diabetic gastroparesis. In addition, there are several noteworthy lessons from these cases. To begin with, there appeared to be a clear correlation between the histological changes noted and the clinical course of diabetes, perhaps indicating the importance of poor glycemic control in their development. The prevalence of delayed gastric emptying correlates weakly with the duration of diabetes [34]. Related factors, such as extreme hyper- or hypoglycemia, even if short-lived, may be even more important as both conditions have been shown experimentally to cause neuronal injury and death (although hyperglycemia has not been shown to affect ICC in an animal model) [31,35,36]. It is therefore possible that patient 1 was spared major changes in gastric pathology because she experienced a shorter and metabolically more stable course of diabetes than patient 2. The only finding of note in the tissue from this patient was a slight decrease in TH immunoreactivity in the circular muscle layer but not in the myenteric plexus. Since the extrinsic nervous system does not directly innervate smooth muscle, these fibers could represent nerves traversing their way to the submucosal plexus. Alternatively, these could also be processes from intrinsic neurons as there is a subset of enteric nerves that also expresses TH [37]. Regardless, the clinicopathological implication of this finding remains unclear.

More remarkable than the above observation, however, was the fact that the changes in the stomach wall appeared to have no relationship to clinical symptomatology. Thus, despite comparable clinical severity, the two patients had dramatically differences in gastric pathology, with one being essentially normal and the other displaying a spectrum of abnormalities affecting nerve, muscle and ICC. These findings provide room for several different speculations. First, it is possible that the changes in Case 2 represent "end-stage" pathology resulting from cumulative and repetitive metabolic insults. As such, their major clinical significance may be to predict intractability rather than causation, which is certainly suggested by the failure of this patient to respond to all interventions including gastric electrical stimulation. If this is true, then gastroparesis symptoms themselves may be due to changes that either occur earlier (such as perhaps the slight loss of TH immunoreactive fibers in the circular muscle layer) or are too subtle to be detected by the techniques we have used (e.g. abnormalities in subcellular signaling pathways in the absence of morphological changes, as has been described in gastric muscle from diabetic animals [38].

Secondly, it could be argued that our patients' symptoms involved mechanisms that we did not investigate, such as vagal dysfunction (suggested by the presence of resting tachycardia in both cases). Changes in vagal function have traditionally been implicated in the pathogenesis of gastroparesis, although solid evidence is lacking and the correlation between autonomic dysfunction and delayed gastric emptying is modest at best [2,34]. Finally, it is possible that diabetic gastroparesis is truly a heterogeneous disorder and that while some patients have obvious gastric pathology (as in case 2), others may develop a pathophysiologically distinct form of gastroparesis resulting from circulating autoimmune factors that can cause dysfunction but not disruption of the nerves or muscle, as has been described [39,40].

## Conclusion

In conclusion, we have described a detailed pathological examination of two patients with diabetic gastroparesis. While it is difficult to generalize based on such a small number, these cases do offer some insight into the pathogenesis of gastroparesis and also provide the basis for several important hypothesis for future studies.

## Abbreviations

nNOS: neuronal nitric oxide synthase; HO-2: heme oxygenase-2; ICC: interstitial cells of Cajal; SP: substance P.

## Competing interests

The authors declare that they have no competing interests.

## Authors' contributions

PJP: protocol design, analysis of results and manuscript writing. NP: protocol writing and submission, collection of material. GG: surgical operation, editing of manuscript. HV: collection of data, editing of manuscript. MSL: tissue processing and staining, analysis of results. GF: supervision of tissue handling, staining and troubleshooting technical aspects of the same; anaylsis of results, manuscript editing.

## Pre-publication history

The pre-publication history for this paper can be accessed here:


